# Family trouble: Heteronormativity, emotion work and queer youth mental health

**DOI:** 10.1177/1363459319860572

**Published:** 2019-07-24

**Authors:** Elizabeth McDermott, Jacqui Gabb, Rachael Eastham, Ali Hanbury

**Affiliations:** Lancaster University, UK; Open University, UK; Lancaster University, UK; The Proud Trust, UK

**Keywords:** family, LGBTQ+, mental health, youth

## Abstract

Conflict with the family about sexual orientation and gender diversity is a key risk factor associated with poor mental health in youth populations. Findings presented here derive from a UK study that employed an interdisciplinary critical mental health approach that de-pathologised emotional distress and conceptualised families as social and affective units that are created through everyday practices. Our aim was to explore how family relationships foster, maintain or harm the mental health and well-being of LGBTQ+ youth. Data were generated through exploratory visual, creative and digital qualitative methods in two phases. Phase 1 involved digital/paper emotion maps and interviews with LGBTQ+ youth aged 16 to 25 (n = 12) and family member/mentor interviews (n = 7). Phase 2 employed diary methods and follow-up interviews (n = 9). The data analytic strategy involved three stages: individual case analysis, cross-sectional thematic analysis and meta-interpretation. We found that family relationships impacted queer youth mental health in complex ways that were related to the establishment of their autonomous queer selves, the desire to remain belonging to their family and the need to maintain a secure environment. The emotion work involved in navigating identity, belonging and security was made difficult because of family heteronormativity, youth autonomy and family expectations, and had a stark impact on queer youth mental health and well-being. Improving the mental health of LGBTQ+ youth requires a much deeper understanding of the emotionality of family relationships and the difficulties negotiating these as a young person.

## Background

We know from psychological research that the family is significant to the mental health of LGBTQ+^1^ youth. Research indicates a clear link between negative family experiences and poor mental health in young LGBTQ+ people (Grossman et al., 2011; Ryan et al., 2009; Simons et al., 2012). D’Augelli et al. (2005) found a strong association between suicidality among LGB youth and parental mistreatment and abuse. Studies have shown that family rejection is a significant predictor in reported levels of depression and suicidal thinking in LGB youth (Ryan et al., 2009). Lack of family support is also a risk factor for suicide in LGB young people (Needham and Austin, 2010).

In contrast, family acceptance and parental support have been found to protect the risk of poor mental health in LGBTQ+ young people (Espelage et al., 2008; Ryan et al., 2010). In a systematic review of parent influences on LGB youth health and well-being, Bouris et al. (2010) found that the emotional dimension of the parent–child relationship, such as support, caring, connectedness and conflict, influenced LGB youths’ mental health. Across studies, a supportive and caring parent–child relationship has emerged as an important correlate to good mental health (Needham and Austin, 2010; Ueno, 2005; Van Beusekom et al., 2015). These patterns and associations are similar for trans and gender diverse youth (Katz-Wise et al., 2018; Simons et al., 2013). Furthermore, positive family environment has also been found as a protective mechanism against LGB discrimination (Freitas et al., 2016). Although this is not a consistent finding with some studies showing that family support was not a protective factor against victimisation in LGB youth (Stettler and Katz, 2017).

However, this evidence is limited and generally tells us that youth whose parents respond well to LGBTQ+ disclosure have better mental health than those whose parents reject or are hostile to a disclosure (Stettler and Katz, 2017). The research has mainly focussed on parental–child relationships and other significant relationships (e.g. friends, aunts, extended family), and factors such as ethnicity, disability and socioeconomic status have had less attention paid to them. There is still much we do not understand about why family relationships impact LGBTQ+ youth mental health. In our view, existing research limits our understanding in three ways: (1) by employing a biomedical framework that conceives of emotional distress as an indication of individual pathology, (2) the under-theorisation of ‘family’ and ‘youth’ and (3) the dominant use of quantitative survey methods which reduces very complex relationships, meanings and emotions to discrete variables (McDermott and Roen, 2016). These methods are important for establishing relationships between factors and capturing the scale of the problem, but they are less able to explain *why* and *how* family relationships influence LGBTQ+ youth mental health.

The approach taken in this study was to de-pathologise emotional distress and conceptualise families as social and affective units that are created through everyday practices. Contemporary social science research has effectively debunked any unitary basis of ‘the family’ (Gabb, 2008). The family as a monolithic, biologically constituted institution fails to accurately represent the diversity of family forms. We conceptualised families as affective spaces of intimacy within which meanings and experiences are constituted by family members in a historical sociocultural context rather than in accordance with naturalistic understandings of reproductive and/or socialisation function (Smart, 2007). In other words, families are envisaged as relationships materialised through sets of practices rather than as a consequence of fixed social categories (mother, father, sibling, etc.) (Morgan, 1996).

Our theoretical framework also considered family relationships as emotive and ‘troublesome’ (Ribbens McCarthy et al., 2013) but important to all young people, regardless of sexuality or gender, because family can provide bonds of belonging, love, support, intimacy, care, safety, food and protection. Wyn et al. (2011) argue that the family has become more relevant as a site of connection and security because young people live in an increasingly uncertain and fragmented world. Bessant et al.’s (2017) analysis of five different countries (United Kingdom, United States, Australia, France, Spain) demonstrates that this insecurity or precarity has been largely precipitated by the evaporation of resources in society to support the transition from childhood to adulthood (such as employment, training, education, homes and finances). Young people in these countries face an increasing burden of deprivation, inequality and disadvantage compared to those over 35, and this impacts their welfare and well-being (Bessant et al., 2017; Standing, 2014). Family support, resources and contact are perhaps more important than ever because of the precarious nature of young people’s lives (Edwards et al., 2014; Smart, 2007). However, the family as a site of security, identity and belonging has always been potentially problematic for young queer people because they may face hostility towards their sexual or gender diversity from family members.

Our research sought to understand the impact of family relationships on LGBTQ+ youth mental health and well-being from a *relationship-oriented perspective*. We focussed on young people’s emotional experiences of connection to, ambivalences with, and isolation from, family relationships. We were interested in the way emotions and relationships were intertwined in the process of managing non-heterosexual sexualities and gender diversity within families and the impact on mental health and well-being. In the next section, we outline our interdisciplinary critical mental health approach that de-pathologises emotional distress. The subsequent section describes the two-phased methodology and then we present findings that suggest family relationships can be troublesome to queer youth mental health because of identity, belonging and security.

## De-pathologising emotional distress in youth mental health research

The medicalisation of human misery and suffering puts the difficult subject of disruptive emotions, feelings and thoughts into the hands of psychology and psychiatry. In this biomedical framework, emotional distress is conceived as an indication of psychological abnormality requiring diagnosis and treatment. The problem for psychiatry is that it has never been able to reliably identify causality, pathology or aetiology of mental dysfunction (Boyle, 2011; Busfield, 2011; Rapley et al., 2011). A key weakness of the discipline is that there are very few visible markers of the ‘disorders’ it attempts to treat (Pilgrim, 2014). Mental health symptoms are subjective descriptions of feelings and emotions (e.g. I have lost interest, I am anxious, I am worried), rather than visible, physical symptoms (e.g. damaged blood vessels, clots, lumps, swellings, heightened immune indicators). Many of the phenomena identified as mental illness within psychiatric diagnostic criteria are regular emotional reactions to life’s difficulties. The subjective experiences of pain, sorrow, elation and misery occur without any necessary implication of pathology (Fernando, 2010).

Mainstream psychiatric and psychological conceptualisations of adolescent mental disorder similarly focus on individual psychological abnormalities as the primary source of distress (Boyle, 2011). The research examining the role of the family in relation to LGBTQ+ youth mental health has largely been conducted within a psychological paradigm utilising the Minority-Stress explanatory framework (Meyer, 2003). In this model, stigma and discrimination specifically related to being a sexual minority make LGB populations vulnerable to poor mental health. The model distinguishes between two types of minority distress: distal processes that are external, objective stressful events and conditions (Meyer, 2003: 676), and proximal processes that are subjective, related to the internalisation of negative sexual attitudes and concealment of identity. This model has also been adapted to use with trans populations (Hendricks and Testa, 2012). As the field of research has developed, psychologists have taken an interest in developing this model and asking what are the mechanisms linking stigma-related stress and mental health problems in sexual-minority youth. Emotional regulation (ER) has been highlighted as one possible mechanism (Hatzenbuehler, 2009; Stettler and Katz, 2017). ER is conceptualised as processes that change an individual’s emotional experience that may be considered adaptive or maladaptive such as rumination, concealment and catastrophising.

Undoubtedly, emotions are crucial to understanding how experiences of being young and queer translate into distress and poor mental health. However, the concept of ER keeps young people’s emotions reduced to diagnostic ‘problem’ categories such as ‘concealment’ and ‘rumination’, and are thus ‘contained’ within a psychomedical rationalist paradigm (McDermott and Roen, 2016). For example, Stettler and Katz (2017) state, ‘identifying whether deficits in ER are major risk factors for psychopathology in GLB youth may help guide clinical interventions with these adolescents’ (p. 385). Investigations of ER are framed by individual psychopathology – a deficit model – that is happening solely in an individual’s mind. There is no discussion that working on emotions we experience may have a *social* component, that our relationships, expectations, employment, material circumstances might impinge on how we manage our emotions.

Significantly, sociologists and cultural theorists have demonstrated that the emotions we experience are shaped partly by social norms (Ahmed, 2004, 2010; Bendelow and Williams, 1998; Hochschild, 1979; Lupton, 1998). Hochschild (1979) asserts that feeling rules are social norms that tell us what, when, where to feel and how long and strongly we can feel. As Hochschild (1979) suggests, not only are emotions and feelings influenced by the social mores of the day, but what we ‘do’ with our emotions or our response to our feelings has also been shown to be governed by these social and cultural norms. If we feel failure or disappointment, this is in relation to perhaps an idealised event such as a wedding. Both Hochschild ([1983] 2003) and Ahmed (2010) use the heterosexual wedding to illustrate the way we ‘manage our hearts’ and how happiness is socially proscribed. Ahmed (2010) states, ‘Disappointment can be experienced as a gap between an ideal and an experience that demands action’. In experiencing an inappropriate emotion – a disappointed bride on their wedding day – this prompts some sort of emotion work, that is, the conscious act of trying to influence the degree/quality of a feeling. The rational individual is expected to discipline their emotions in order to experience the appropriate emotion at different times and places (Elias, 1994; Rose, 1999).

The control of emotions is central to normative developmental discourses that define adults as mature and able to control their emotions, and youth as emotionally immature and unable to control their emotions (McDermott and Roen, 2016; Lesko, 2001). The underlying message is that young people’s ‘disorderly’ emotions are not to be taken seriously; they are a product of hormonal changes and a common phase of the adolescent years that will diminish over time (McDermott and Roen, 2016). The tendency for young people’s emotions to be temporalised (‘it’s a phase’, ‘they will grow out of it’) serves to reproduce a hierarchical division between the rational adult and the emotional adolescent (Burman, 2008; Lesko, 2001). In this study, we de-pathologise emotional distress and argue that to understand how family relationships contribute to LGBTQ+ youth mental health and well-being requires a focus on the ways in which young people embody, negotiate and manage emotion, and the social, economic and cultural familial context in which this takes place. In the next section, we describe the emotion-centred methodology we employed to answer the research question: ‘In what ways do family relationships impact on LGBTQ+ youth mental health and well-being?’

## Methodology

Researching young people who are marginalised by their age, LGBTQ+ status and mental health, and asking about experiences of family relationships and mental health, constitutes a ‘hard-to-reach’ population and a ‘hard-to-talk-about’ topic. Our methodological approach recognised that ‘youth’ occupy a distinct and precarious social position that is controlled through specific policy and legislation (e.g. age of consent laws, compulsory schooling) and often positions them as powerless in relation to adults (Heath et al., 2009; Sime, 2008). Consequently, what youth research participants disclose can put them at risk of censure, objectification and surveillance (Cahill, 2007).

Traditional qualitative methods such as interviewing can represent an adult centred, top-down approach to data collection (Drew et al., 2010) that privileges the verbal articulation of experience and provides limited access to the emotional dimensions of young people’s lives (Bragg and Buckingham, 2008). Consequently, we employed visual, creative and digital qualitative methods to facilitate youth inclusivity and value their agency and ‘ways of knowing’ (McDermott, 2015; McDermott and Roen, 2012; McDermott et al., 2013). This was particularly important because we were generating ‘sensitive’ data on experiences that could be stigmatised or distressing (e.g. family rejection, homo/bi/transphobia, self-harm) (Meezan and James, 2009; Panfil et al., 2017). Our critical mental health framework put emotions at the centre of the research process, but emotions are often difficult to express through words (Mays et al., 2011; Sime, 2008). We designed a study using visual, creative and digital qualitative methods to facilitate the capture of different and non-verbal dimensions of emotional experience (Bagnoli, 2009; Copeland and Agosto, 2012; McDermott et al., 2017).

### Ethics

Asking youth about their sexual orientation may place them at risk from discrimination (e.g. homophobic bullying) or harm (e.g. emotional distress) and/or be sensitive because they are undecided, confused and/or apprehensive about their LGBTQ+ status and/or their mental health (Elze, 2009; McDermott and Roen, 2016). Anonymity was ensured through recording interviews using an encrypted digital recorder, and transcription was undertaken by a transcriber who had signed a confidentiality agreement. All identifying features were removed from the data. All anonymised data were stored electronically on a password protected secure drive on a university server. All original data were deleted once the anonymised versions of the data had been created. Paper consent forms and visual data were kept in a locked filing cabinet in the locked office of the principal investigator. We collaborated carefully with participants to ensure their safety and anonymity were guaranteed. In one case, for example, we agreed to use abstract/opinion-based quotes only in publication, as the participant was extremely concerned about being recognised. Recruitment and all face-to-face meetings took place exclusively through LGBTQ+ organisations so participants had consistent access to support services via telephone, Internet or face to face, and could easily and immediately access help if required.

Informed consent was gained through written or electronic signatures from all participants (participants were 16–25 years old and parental consent is not required) before the research commenced and verbal consent was sought at intervals throughout the study (McDermott et al., 2016). Face-to-face interviews were recorded using an encrypted digital recorder. All identifying features were removed from all data which were stored electronically on a password protected secure drive on a university server or in a locked filing cabinet. All data were anonymised and participants were ascribed pseudonyms. The research received full ethical approval from the Faculty of Health and Medicine, Lancaster University ethics committee.

### Method

This study utilised visual, creative and digital qualitative methods in two phases. Phase 1 involved (1) face-to-face and online semi-structured interviews and family mapping with LGBTQ+ youth (n = 12), and (2) face-to-face and online semi-structured interviews with ‘family members’ (n = 7). Phase 2 involved LGBTQ+ youth keeping a week-long diary and follow-up interview (n = 9). All participants had to be over 16 years of age, living in England and have the capacity to give consent. Youth participants’ eligibility included self-definition as LGBTQ+ and aged between 16 and 25 years; family members were eligible if they self-identified as family, or family-like to an LGBTQ+ young person aged 16 to 30 years old. Both phases used a purposive recruitment strategy (Patton, 1990) with specific attention paid to ethnicity and socio-demographic status. Participants were recruited via LGBTQ+ youth organisations in England using paper/digital flyers and face-to-face contact. Tables 1 and 2 show the sample demographics for both phases of the research.

**Table 1.** Young people participants.

	N = 13
Age groups	
17–19 years	4
20–25 years	9
Sexual identity	
Lesbian	3
Gay	4
Bisexual	5
Pansexual and queer	1
Other	0
Gender identity	
(Cis) female	6
(Cis) male	3
Trans female	1
Trans male	1
Other	2
Ethnicity	
White British	8
Black and minority ethnic	4
White (Other)	1
Disability	
Yes	6
No	7
Free school meals	
Yes	4
No	9
Unsure	0
Parent/carer university degree	
Yes	7
No	6
Unsure	0
Self-harm	
Yes	10
No	3
Suicidality	
Suicidal thoughts	6
Suicidal plan/attempt	6
No	1

**Table 2.** Family member participants.

	N = 7
Relationship to LGBTQ+ Young People	
Family member	5
Mentor	2
Sexual identity	
Lesbian	2
Gay	1
Bisexual	1
Pansexual and queer	1
Heterosexual	2
Gender identity	
(Cis) female	6
(Cis) male	1
Trans female	0
Trans male	0
Other	0
Ethnicity	
White British	5
Black and minority ethnic	1
White (Other)	1
Disability	
Yes	1
No	6

### Data collection phase 1

The aim of phase 1 was to collect LGBTQ+ youth and family/family-like perspectives, experiences and emotions about family relationships and their impact on LGBTQ+ youth mental health and well-being. The semi-structured face-to-face and online interviews with LGBTQ+ youth (n = 12) were facilitated by a ‘toolkit’ of visual activities and an interview schedule. The map-making structured and prompted the discussion and minimised the pressure on participants in the face-to-face research encounter. To capture emotion, we adapted emotion mapping techniques (Gabb and Singh, 2015; Gabb, 2008) and used emoticon stickers to reflect their feelings about different family relationships. We also used ‘scenario stickers’ to stimulate discussion about the characteristics of different relationships within their families, asking, for example, which family member would they be most likely to ask to borrow money for a bus to youth group. The interview schedule had four sections focussed on family relationships, social support, sexuality and gender, and mental health and well-being. At the end of the interview, participants completed a short demographic questionnaire. The semi-structured face-to-face/online interviews with family members (n = 7) used an interview schedule to structure discussion about their relationships with their respective LGBTQ+ young person. The schedule had four sections focussed on family relationships, young person’s coming out, managing LGBTQ+ identity within the family, and mental health and well-being. These participants also completed a short demographic questionnaire.

### Data collection phase 2

The aim of phase 2 was to capture immediate, everyday practices and emotions of LGBTQ+ youth. Youth participants (n = 9) kept a diary of family interactions over 1 week in a format of their choosing – paper or online. Diary methods offer a privacy, which can be effective for sensitive topics (Gabb, 2008). Participants were asked to answer five questions each day such as ‘Who from your family have you seen/had contact with today? How did these interactions make you feel?’ As with phase 1, participants used emoticon stickers to capture their feelings about events they wrote about. Participants also completed a daily single subjective mental health and well-being question (Office for National Statistics Personal Wellbeing Domain for Children and Young People). After the diary was complete, participants had a follow-up face-to-face or online unstructured interview that explored the meanings of the interactions captured in the diary.

### Data analysis

All face-to-face interviews were transcribed, and the online interviews electronically archived. Family maps and diary data were anonymised and scanned into a digital format, and all materials were inputted into the data analysis software NVivo. There were three stages to our data analytic strategy. First, we conducted a case analysis for the data we had for each individual youth participant (demographic, interview 1, map, diary, mental health questions, interview 2, field notes) (Miles and Huberman, 1994; Yin, 2014). The case analysis utilised an ‘I-feel’ mapping exercise, drawing on ‘I-Poems’ (Edwards and Weller, 2012), to trace how a participant spoke about their feelings/emotions in relation to their family. We then designed a case analysis question template (Miles and Huberman, 1994) to interrogate how the participant orientated to their family, LGBTQ+ identity and emotions/mental health.

The second stage of the data analysis strategy was a cross-sectional approach to the entire dataset. Guided by the research questions and the case-study analysis, a coding framework was developed by three members of the research team to improve inter-coder validity and reliability (Braun and Clark, 2006). The descriptive (e.g. age), interpretative (e.g. frivolity) and theoretical (e.g. heteronormativity) codes that resulted were applied across the whole dataset, including the family member data. Subsequently, the project team conducted a thematic analysis of data in each of the cross-sectional codes (Mason, 2002). The third stage of the data analysis strategy involved a meta-interpretation to develop relationships between the cross-sectional code data analysis (e.g. emotion work, family practices of communication) within our theoretical frame to answer the research question, ‘How do family relationships impact queer youth mental health and well-being?’ We present, in the following three sections, these findings that suggest family relationships can be troublesome to queer youth mental health.

## Happy families: belonging, security and becoming

In response to the direct question about how family relationships impact their mental health, all participants were quite clear that family was very important to theirs and other young people’s mental health. Participants stated there was something specific about family support that was qualitatively different from other sources of support such as friends. Hannah (Lesbian, unsure-female, White British) stated,

I think it does, I think it has more of a, because it’s your support network at the end of the day. You have your friends support network but it’s the support network that you are born with, hopefully, and that’s the most important, not the most. I think it impacts it in ways even if you don’t realise it.

Similarly, Melissa (bisexual, Cis-female, Black, Asian & Minority Ethnic (BAME)) explained,

I think at first I was really upset because I was like this is my mum, she’s kind of meant to love you no matter what you do.

Hannah and Melissa had different relationships to their families and this is reflected in their answers to the interview question. Melissa left home because of her family’s attitude to her sexuality. Hannah has a better relationship with her family; she has disclosed her sexuality (and mental health) but this was quite fraught emotionally. Family members gave examples of ‘unconditional’ relationships with their LGBTQ+ young people, and Katie (heterosexual, Cis-female, White British) described this explicitly in relation to her sister’s LGBTQ+ identity:

We have always been told that it honestly doesn’t make a difference to us whether you are gay, whether you’re straight, whether you are bisexual, it doesn’t matter. Happiness is what matters […] I know I could tell my parents anything and they would still love me.

As this suggests, our participants described that the ‘ideal’ family should feel supportive, caring and close and provide an unconditional love where you are looked after, feel safe and can be happy. These views were drawing on cultural expectations that biological family *should* be a site of love, happiness and positive family relationships (Gabb, 2008; Ahmed, 2010). Being able to explore their sexual and gender identity in a safe environment and *simultaneously* remain bonded to their families was very important to their mental health and well-being (Gabb et al., 2020). Josh (gay, Cis-male, White British) emphasised the importance of some of his family accepting his sexuality and staying connected to them despite his mother’s homophobia (she threw him out of home):

Everything’s changed with these because they accept who I am, and they are not fazed by it. Like my sister, my brother and my dad – I’m still me. I am still the happy go lucky chavvy lad that they brought up, the one that fights and gets in trouble and I’m still me.

Jamie (bisexual, trans male, White British) described how he would have liked his family to respond to his trans identity:

yesterday I went to see [FILM] at the cinema the new LGBT film and there was this part where, spoilers by the way, where the mother was being like really supportive of him and he like started crying; it was all really sweet and then I started crying like why did I never get that; why did I never have a big speech about the fact that I won’t change to them and I’m still accepted in the family.

Unfortunately, the experience of recognising themselves as sexually and/or gender diverse often meant our participants felt there was a tension in their family life. Our data suggest the ongoing process of becoming an autonomous queer individual that did not fit with heteronormative family expectations created difficulties/tensions in belonging to their families, and for some it threatened a secure and safe home and was damaging to mental health.

## Family trouble: heteronormativity, autonomy and expectations

The majority of the participants when speaking about their mental health made a direct link between their LGBTQ+ identity and their family’s attitudes to sexual and gender diversity. Chris described the negative impact of a hostile family environment:

It’s like with my family I knew I didn’t like the constant insults […] and with casual homophobia I think that ended up impacting my mental health quite a lot and not being to be as open meant that I was very inwards which I think ended up just exasperating the whole mental health problems. (Pansexual, Other male, White British)

In contrast, Hannah explained how openness within the family improved her depression:

I don’t know whether it’s linked or whether its not linked but when I came out to my dad, maybe a month after I just started feeling [PAUSE] better and have not really had any problems since, any major episodes since. (Lesbian, unsure female, White British)

The data from the participants demonstrated a consistent presence of homo/bi/transphobia from across a range of family members – sister, brother, granddad, mum, dad, uncle, aunt, extended family. Our dataset had plenty of incidents of homo/bi/transphobia, and the participants were well aware of who in the family were hostile to LGBTQ+ people. For some participants, this hostility resulted in being thrown out of home and for others they left of their own accord because they were no longer able to manage the situation. The emotional difficulties of navigating heteronormativity within the family – trying to judge what to say, who to tell, who to hide from – had a direct impact on the participants’ mental health and well-being. These problems were often compounded by young people’s unequal power relationships with the adults in their family. Young people lacked autonomy and felt they had to comply with a raft of family expectations in relation to education, employment, religion, culture and ethnicity. Melissa (bisexual, Cis-female, BAME) described how trying to be a ‘good’ daughter made her unhappy:

I think it was because when I was younger I tried to do everything I could to like not impress her, but just like make her happy in a way. Everything that she wanted me to do to be a good daughter, I suppose. But then I got to the point where I was like either I’m going to be really unhappy, we’re both actually going to be really unhappy or I can try to make myself happy.

Melissa ‘failed’ to comply with her mother’s expectations because she was bisexual, stated she was not getting married or having children, refused to observe religious customs and had not pursued her mother’s expected education and employment pathway. Her ‘disobedience’ created a very stressful family home, which contributed to Melissa’s mental health problems. Similarly, Jamie (bisexual, trans male, White British) described the strain of being trans, the weight of expectation on young people and having limited autonomy with the family:

Its stressful being LGBT because society can just screw you over sometimes. It is especially stressful being trans because you can’t escape a situation where you don’t have, where you don’t announce your transgender. It’s stressful being a young person in today’s society because there is a lot of pressure on young people. You have to get a job, you have to work towards getting a job, you need all these qualifications otherwise you can’t get into college and can’t get a job. Then it is finally very, very stressful to function in a family unit because there is a lot of responsibility on you to please your parents.

Within a complex negotiation of regulatory heteronormative discourses and relationships, economic dependency, cultural expectations and material constraints, the participants in our study were not ‘rebelling’ but, instead, struggling for autonomy to become their sexual/gendered young adult selves. Autonomy is commonly understood to be important to developing into a rational and mature subject, and it is defined as a growing independence from parents and carers with free will to act (Walkerdine et al., 2001). The problem for some queer youth is that their struggle for autonomy takes place in circumstances which can be hostile, where life is dependent on adults. This creates a tremendous pressure and conflict between wanting to be a mature, autonomous queer young person but wanting to belong to the family and remain in a secure setting (McDermott and Roen, 2016). For queer youth, their transgression of heteronormativity, not fulfilling family expectations, and lack of autonomy can threaten their security and family connection. Our data analysis suggests that the emotions required to decipher sets of ‘paradoxical family practices’ (Gabb et al., 2020) required young people to navigate family relationships that did not fit a standard norm. Making sense of family-specific relationships and surviving stressful family settings were significant to mental health and well-being.

## Emotion work and queer youth mental health

As indicated in the previous sections, family relationships were important to the youth participants and they worked hard to maintain their familial bonds. Our analysis suggests it is the emotion work involved in this relationship maintenance, endurance, repair and re-negotiation that is key to explaining why family relationships are so influential to queer youth mental health and well-being (Gabb et al., 2020). In this study, we had a specific critical mental health framework that put emotions at the centre of conceptualising young people’s mental health. The research team was still surprised by the intensity of the emotions in the data, from young people and family members, when explaining their LGBTQ+ status, family relationships and mental health. In addition to intensity, it was the extent that young people were managing, coping, reacting, changing and adapting their emotions and their responses to their emotions. Participants specifically described this as ‘Stiff upper lip’ (Chris); Coping with ‘a weight’ (Emma); ‘Carrying a weight’, ‘Side stepping’ (in conversation), ‘On guard’, ‘Bite my lip’ (Hannah); ‘Grew a thicker skin’, ‘Just deal with it’, ‘Grit your teeth and bare it’(Jamie); ‘Take it on the chin’, ‘Brave face’ (Josh); ‘Stop trying’, ‘Shut down’, ‘Get used to it’ (Melissa); ‘Detachment’, ‘Stop feelings’; ‘Act fairly normal’ (Skye).

In Table 3, we characterise the forms of emotion work expressed by the participants. We do not mean these to be a typology of ‘emotion work’, but they serve more to demonstrate the emotionality of the strategising, thinking, managing, feeling, required to survive family life when you are young and queer. We also think this shows the burden on mental health. We do not want to repeat the psychopathologising of queer youth emotional distress, or suggest particular emotion work strategies lead to particular mental health problems. In contrast to Hochschild (1979), our view is that the participants’ emotion work cannot be ‘chopped up’ in meaningful ways as the modes typically overlap and can be temporal, and would prove reductive and limit our understanding of the extent of emotion work being undertaken by the participants.

**Table 3.** Characterisation of queer youth emotion work.

**Emotion work**:
Withdrawal (withholding, silence)
Masquerade (hiding, secrecy)
Avoidance (walking out, detachment)
Rationalising (deal with it)
Surveillance (steering, deflecting, humour)
Resistance and refusal

Our analysis does suggest, however, that participants’ emotion work is crucial to understanding the relationship between families and queer youth mental health. Most importantly, this emotion work is agentic, and not a sign of psychopathology or maladaptive ER. If we take ‘withdrawal’ as an example, this was emotion work we recognised in our analysis where young people described keeping silent about their emotions and making no emotional demands on family relationships. In our data, this was a common strategy where young people described withdrawing, both temporarily and permanently, because it seemed like the only way to deal with confusing, hurtful, unnamed feelings and relationships – often related to their queerness. So withdrawal or isolation is agentic not imposed by outside world nor is it a sign of psychopathology or maladaptive ER. It is a reasonable survival strategy to employ when living in a hostile environment with few resources, little autonomy and no economical independence. For example, Melissa (bisexual, Cis-female, BAME) states regarding her mum:

if I don’t live in the same house as her she’s not always like [PAUSE] policing what I’m doing, and when she does that I get more irritated and I just shut down even more and I just stop talking to her about anything and it just gets worse and worse.

This contrasts with young people in families where they did not need to withdraw because family members were invested in supporting them and understanding their emotional distress. In the interviews with family members, Lisa (Lesbian, Cis-female, White British) spoke of efforts to understand the potential root of her daughter’s anxiety and support her using mental health services. Similarly, Mark (gay, Cis-male, White British) mentioned his own mental health issues too while considerately reflecting on how this interplays with and impacts those of his daughter.

However, for many of our participants, ‘doing’ emotion work and maintaining the ‘happy family pretence’ was a survival strategy that enabled some to remain housed, fed and safe. The precarious nature of young lives was evident in our participants’ concerns about security, housing and finances, and some reflected how they could not afford *not* to do emotion work of some sort. For example, Kelly (bisexual, Cis-female, BAME) had been in care and was estranged from her family home, with very limited resources and stated she was ‘used to feeling terrible’. Homeless Josh (gay, Cis-male, White British) deployed a ‘brave face’ to get on with in his current circumstances. In both cases, the imperatives to engage in emotion work were for the purposes of basic survival, and these were often detrimental to their mental health.

## Conclusion

The findings from this study suggest that queer young people’s mental health is deeply affected by their relationships with their families but in complex ways. Our findings indicate that while the disclosure of sexual and/or gender diversity to family members is crucial to good mental health, it is the emotionality of family relationships, and queer youth negotiation of these, that is important to recognise when trying to understand why and how family is so influential of queer youth mental health. It is the meaning – socially, culturally and economically – of ‘paradoxical family practices’ (Gabb et al., 2020) that matters.

Through the use of an interdisciplinary critical mental health approach that conceived emotional distress as a regular capacity of humans to feel, and utilised sociological theorisations of family, youth, sexuality and gender, we employed a creative, emotion-centred methodology to capture intricate relationships, meanings and emotions. As a result, our analysis centred queer youth within the powerful and emotional dynamics of family life to investigate their mental health.

Youth is a central concept in this project, and it has been young people’s position within the family which has perhaps been most forcefully ‘present’ throughout our analysis. The power dynamics between young people and the adults in their family are usually absent from investigations of LGBTQ+ youth mental health, youth is conceived as an age on a development spectrum and family relationships are categories of biological kin (mother, father, etc.). What has ‘pressed’ upon our analysis, through our engagement with all the participants (youth and family), has been the difficulties youth have negotiating family life because of their age, sexual and/or gender diversity, ethnicity/religion and economic dependency. The emotion work involved in becoming their autonomous queer young adult selves, in becoming who they felt they were, but remaining connected to family as a site of identity, love and sometimes just for safety, was intense and often overwhelming, and this compromised their mental health and well-being.

Despite these difficulties, the young people in our study showed an agentic intent, competency, self-awareness and extensive compassion to family members, which, we think, intensified the emotion work involved in maintaining their family relationships. In other words, because they loved, respected, were grateful and cared for their family members, they tried hard to remain connected and belong. Similarly, where there were positive family relationships, this was nearly always where family members gave time, respect and space for the young person to develop their autonomy and self-determination in a supportive and communicative environment. Unsurprisingly, it was these types of relationships that promoted queer youth well-being.

Alongside the power differential between youth/adults, heteronormativity imbued family relationships that made them often oppressive, hostile and controlling. Levels of heteronormative surveillance, scrutiny and policing by family members increased levels of emotional distress in the young people and in some circumstances meant they left or were forced to leave their family home. Our analysis showed quite clearly that within a complex negotiation of regulatory heteronormative discourses and relationships, economic dependency, cultural expectations and material constraints, the participants in our study were not ‘rebelling’ but, instead, struggling for autonomy to become their sexual/gendered young adult selves. The emotion work involved in this struggle had a direct negative impact on youth mental health.

Within our analysis, we could not avoid the precarious position of young people and this was especially acute for those who were poor and/or BAME. The site of the family as the only major resource in the lives of most of our participants underscores the ways that the navigation of family heteronormativity can intensify queer youth’s precarious position. The transgression of heteronormative family expectations has potentially dire consequences for queer youth if their family is the only resource that is available to provide shelter, food, love and care. Taking an intersectional viewpoint, it was evident that multiple layers of inequalities can compound challenges; in other words, queerness can amplify the precariousness of young poor or Black lives.

The study is limited by its size and while non-probability samples of LGBTQ+ youth allow the study of important health issues, it is difficult to determine whether findings are characteristic of the population in general or solely the sample recruited. Longitudinal prospective studies of cohorts of LGBTQ+ and non-LGBTQ+ young people that compare family and mental health would help explain the relationships identified in this study as well as other (as yet unrecognised) factors. Our findings are embryonic, but the study contributes theoretically, methodologically and empirically to developing a future frame of investigation that de-pathologises emotional distress and disrupts tenacious stereotypes of young people as over-emotional and ‘out of control’. Our findings suggest that tackling queer youth mental health and promoting well-being might require a different angle of intervention, one that is non-clinical, away from psychopathology and stigma. Social and psychological interventions must help queer youth and their families better navigate their relationships while understanding their complex emotionality and providing a safety net for queer youth if family relationships break down.

## Notes

1. We use LGBTQ+ to refer to sexual and gender identity because this was the preferred term used by participants. References to other research use the author’s original terminology for sexuality/gender. We use ‘queer’ to indicate a theoretical orientation to Queer Theory and Heteronormativity.
